# MAM and LDL Receptor Class A Domain Containing 1 Deficiency Aggravates Hepatic Fibrosis in Diet-Induced Metabolic Dysfunction-Associated Steatohepatitis

**DOI:** 10.1016/j.gastha.2025.100854

**Published:** 2025-11-29

**Authors:** Jashdeep Bhattacharjee, Linda X. Wang, Brianna Meneses, Juliet A. Emamaullee, Mark R. Frey, Rohit Kohli

**Affiliations:** 1 Department of Pediatrics, Division of Gastroenterology, Hepatology and Nutrition, Children's Hospital Los Angeles, Los Angeles, California; 2 Division of Abdominal Organ Transplantation and Hepatobiliary Surgery, Department of Surgery, Keck School of Medicine, University of Southern California, Los Angeles, California; 3 Department of Cancer Biology, Keck School of Medicine, University of Southern California, Los Angeles, California

Metabolic dysfunction-associated steatohepatitis (MASH) is the second leading cause of liver transplantation in the United States, posing a tremendous economic burden on health care.^1^ Bile acid signaling, specifically farnesoid X receptor (FXR) agonists, has been investigated as a potential target for MASH therapeutics. Downstream of FXR activation, fibroblast growth factor 15/19 (FGF15/19; FGF15 in mice and FGF19 in humans) is secreted into the portal circulation, inhibiting bile acid synthesis and de novo lipogenesis, thereby reducing hepatic steatosis, inflammation, and hepatic fibrosis.^2^ However, due to concerns for side effects such as dyslipidemia, FXR agonists have not received regulatory approval for the treatment of MASH.^3^

Previously, we demonstrated that whole-body *Fgf15*-deficient mice are resistant to a MASH-inducing obesogenic (MIO) diet.^4^ MALRD1 (MAM and LDL receptor class A domain containing 1), also known as DIET1, ostensibly restricted to the intestinal epithelium, regulates the expression and secretion of FGF15/19 from the enterocytes, and variants of *MALRD1* have been shown to alter the expression and secretion of FGF19 from intestinal epithelial cells.^5^ Interestingly, genome-wide association studies have shown associations of single-nucleotide polymorphisms in *MALRD1* with obesity-related traits.^6^^,^^7^ Thus, considering *MALRD1* as a novel and understudied molecule in the bile acid signaling pathway, we investigated the role of MALRD1 in MASH progression using *Malrd1* knockout mice (*Malrd1* KO). Here we report the hitherto unknown presence of MALRD1 in hepatic stellate cells (HSCs) and outline MALRD1's FGF15/19 independent role in HSC-activated transforming growth factor β (TGFβ)-mediated MASH fibrosis.

*Malrd1* KO gained body weight (Figure A1A and B) and adiposity (Figure A1C), unlike the whole-body *Fgf15* KO fed an MIO diet.^4^ Plasma alanine aminotransferase levels in the *Malrd1* KO mice were higher on the MIO Diet (Figure 1A). Interestingly, *Malrd1* KO mice also had lower liver weight and liver-to-body weight ratios than wild-type mice on the MIO diet (Figure A1D and E). This phenotype of a smaller, "shrunken" liver matched the observation that Sirius Red staining of liver sections from *Malrd1* KO mice (Figure 1B). The *Malrd1* KO mice have a higher expression of TGFβ target genes in the liver (Figure 1C) and a higher incidence of advanced-stage fibrosis (Fibrosis Stage > 2) (Figure 1D). The liver RNASeq data showed increased expression of genes (Fold change > 1.5) involved in hepatic fibrosis and HSC activation in *Malrd1* KO mice (Figure A2A and B). Further pathway analysis of hepatic genes (1.5-fold upregulated and downregulated) revealed enrichment of signaling pathways, including the hepatic fibrosis signaling pathway (Figure A2C) in the *Malrd1* KO mice. Spatial transcriptomics using paraffin-embedded liver blocks from the Sirius Red fibrosis analysis revealed differential gene expression, with 18 distinct clusters found through neighborhood analysis (Figure 1E and F). Based on gene expression in liver sections, *Malrd1* deficiency induces metabolic stress pathways, such as ferroptosis and oxidative phosphorylation, which are known to be aggravated by TGFβ^8^ (Figure A2D). Since we observed a higher incidence of hepatic fibrosis and activation of the hepatic fibrosis signaling pathway in the livers of *Malrd1* KO mice, we investigated the presence of MALRD1 in HSCs, a key player in the development of TGFβ-driven hepatic fibrosis. We confirmed the presence of MALRD1 in primary HSCs isolated from the wild-type mice livers (Figure 2A). Furthermore, we observed an increased expression of *Malrd1* in T0688 (immortalized murine HSC) cells treated with recombinant transforming growth factor-β1 (Figure 2B and C).

**Figure 1:**
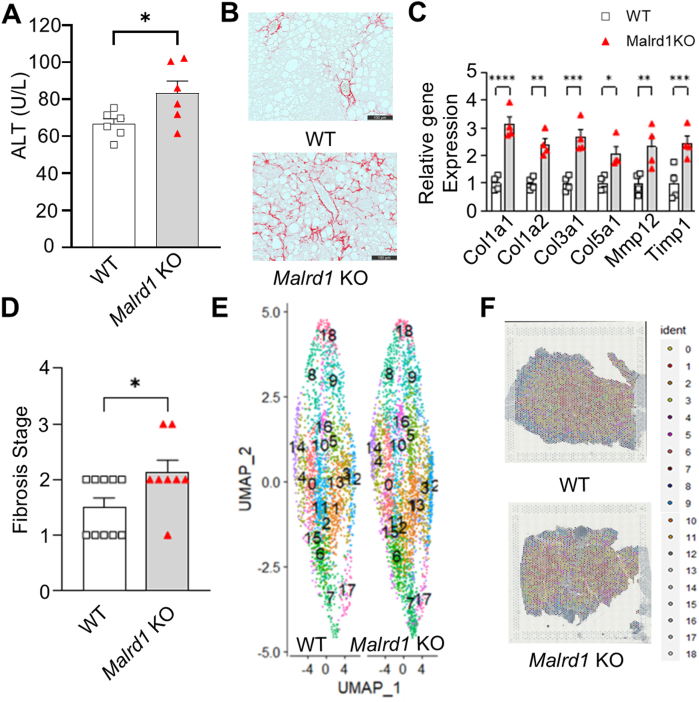
*Malrd1* KO mice exhibit higher Alanine transaminase and aggravated hepatic fibrosis. 6–8 weeks old male wild-type (WT) and *Malrd1* KO mice fed a MASH-inducing obesogenic (MIO) diet for 32 weeks *ad libitum*. (A) MIO-fed *Malrd1* KO have a higher plasma alanine transaminase (ALT) (U/L) levels than WT mice at week 32, (B) Representative histology image of Sirius Red staining of the liver cross-section from WT and Malrd1KO mice, (C) *Malrd1* KO mice higher expression of transforming growth factor β (TGFβ) target genes in the liver than WT mice, (D) *Malrd1* KO mice group has a higher incidence of advanced-stage hepatic fibrosis than WT mice (*P* < .05), (E) Representative image of spatial transcriptomics neighborhood cluster based on the differential expression of genes, (F) Spatial distribution of the clusters across the liver cross-section of MIO-fed WT and *Malrd1* KO mice. (N = 4-10). t-test or Two-Way Anova. Mean ± SEM. ∗∗∗∗*P* < .0001, ∗∗∗*P* < .001, ∗∗*P* < .01, ∗*P* < .05.

**Figure 2:**
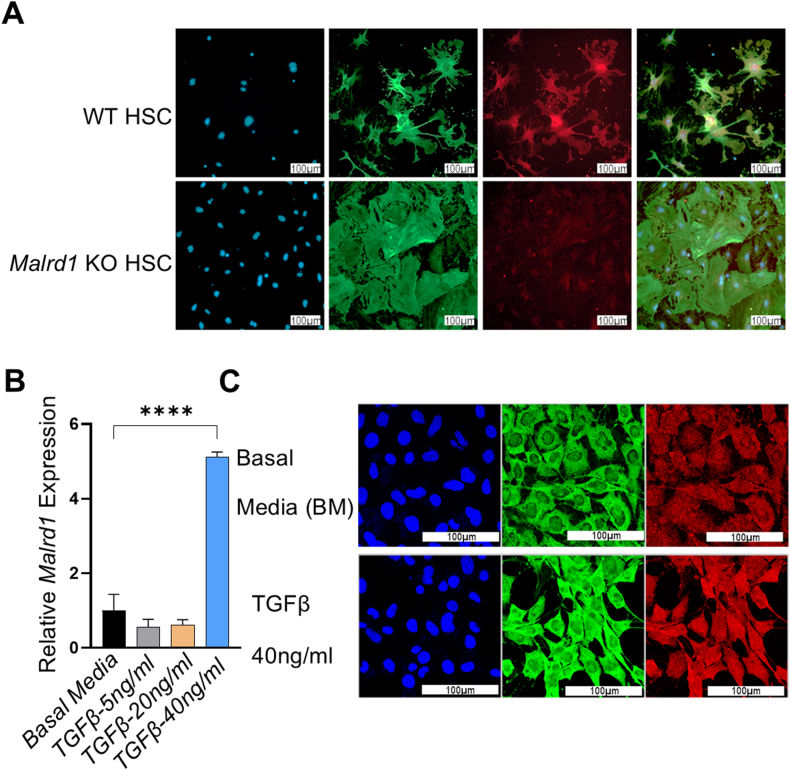
Activated HSCs express MALRD1. (A) Primary murine HSC isolated from WT mice expressed MALRD1 (red) and α-smooth muscle actin (αSMA, green). HSCs from *Malrd1* KO mice express αSMA but not MALRD1. Immortalized murine hepatic stellate cells (T0688) were treated with recombinant murine transforming growth factor-β1 (TGFβ1) at 5 ng/ml, 20 ng/ml, and 40 ng/ml for 6 days. At a 40 ng/ml concentration, T0688 has shown (B) higher expression of *Malrd1* and (C) brighter staining for MALRD1 (red) than T0688 in basal media. Nucleus counterstain DAPI (Blue). (N = 3), One-way Anova, Mean ± SEM. ∗∗∗∗*P* < .0001.

Our findings demonstrate an FGF15/19 independent role of MALRD1 in liver fibrosis, as unlike small intestine-specific *Fgf15* knockout mice,^9^ *Malrd1* KO mice exhibit an obesogenic phenotype with a higher incidence of advanced liver fibrosis. We report for the first time that MALRD1 is present in HSCs and TGFβ1 induces its expression in HSCs. Our data suggests a possible function of MALRD1 in impeding TGFβ-mediated fibrosis signaling, wherein the absence of MALRD1 aggravates the expression of TGFβ target genes, leading to fibrosis, and a higher TGFβ1 concentration induces *Malrd1* expression in the HSC, suggesting the importance of MALRD1 in restricting HSC activation, which is essential for reversing fibrosis.^10^ Since we used whole-body *Malrd1* KO mice, our study could not distinguish the contribution of HSC-MALRD1 versus intestinal-MALRD1 to MASH pathogenesis. In the future, studies using tissue-specific *Malrd1*-deficient mice will provide insight into the molecular interactions of MALRD1 in the HSC during MASH and the influence of intestinal MALRD1 on the action of HSC-MALRD1 during MASH that might lead to the identification of potential therapeutic targets for MASH.

## supplementarary material

Supplementary Material

Extended PDF
