# Supplementary material for: MAM and LDL Receptor Class A Domain Containing 1 Deficiency Aggravates Hepatic Fibrosis in Diet-Induced Metabolic Dysfunction-Associated Steatohepatitis
Source: Gastro Hep Adv. 2025 Nov 29;5(2):100854. doi: 10.1016/j.gastha.2025.100854 (PMC12805096; doi:10.1016/j.gastha.2025.100854)
Supplement: Supplementary Material [file mmc1.pdf]

Materials and methods

All animal studies were reviewed, approved, and monitored by Animal Care and Use Committee at Children's Hospital Los Angeles (assurance#D16-00175). 6-8 week-old male C57Bl6/J wild type (WT) and whole body *Malrd1* knockout (*Malrd1* KO) mice were housed in a 12-hour light-dark cycle maintained in a (22±2˚C) temperature-controlled room. Beginning at 6 to 8 weeks of age, male WT and *Malrd1* KO mice were fed on a MASH-inducing obesity (MIO) diet (D12331i, Research Diets, Inc., 58 kcal % fat; Research Diets, New Brunswick, NJ) and drinking water containing fructose (55% fructose by weight; Acros Organics, Morris Plains, NJ) and sucrose (45% sucrose by weight; Acros Organics, Morris Plains, NJ) mixture at a concentration of 42 g/l[1]. Animals were provided *ad libitum* access to the MIO diets.

Body composition

The body composition of mice (adiposity) was determined using an EchoMRI™-100H Body Composition Analyzer (EchoMRI, Texas, USA).

Plasma alanine transaminase (ALT) assay

Plasma isolated from whole blood was used to measure ALT concentration using the ALT Activity Assay kit (Sigma, St. Louis, MO) according to the manufacturer's instructions.

Liver Histology Analysis

Sirius Red staining was used to evaluate hepatic fibrosis stage (0–4)[2].

qPCR

5µg of total RNA was used to generate cDNA using the SuperScript III First-Strand Synthesis System (Invitrogen, Carlsbad, CA). Quantitative PCR was performed with the Taqman Probe using TaqMan™ Fast Advanced Master Mix (Applied Biosystems, Foster City, CA) in the 7900HT qPCR platform (Applied Biosystems, Foster City, CA). ΔΔCt was used to calculate the fold change of expression of *Malrd1* (PrimerSequence:Forward 5'-CAGAACCCTCAGTAGAAAGGTTAG 3'; Reverse 5' TGGTGGCACTTGTGTGATAG-3',Probe 5' /56-FAM/AGGATGGAC/ZEN/AGGAAATCGATGCCA/3IABkFQ 3'), *Col1a1, Col1a2, Col3a1, Col5a1, Mmp12, Timp1* normalized with the housekeeping gene, *Rpl18* using Taqman probes (Thermo Fisher Scientific, Carlsbad, CA).

RNA Sequencing

5µg of total RNA from the livers of WT and *Malrd1* KO mice (N=5) were sequenced on the Illumina NextSeq platform. The reads were mapped to the UCSC transcript set, and gene expression was estimated using RSEM. Differentially expressed genes were identified with edgeR. QIAGEN IPA was used for pathway analysis.

Spatial Transcriptomics

Spatial transcriptomic profiling was conducted using the 10× Genomics Visium CytAssist on liver sections from one wild-type and three *Malrd1* knockout mice. RNA was extracted with the Qiagen RNeasy kit, and quality was checked using an Agilent BioAnalyzer. Samples were prepared and sectioned onto charged slides, then processed according to the manufacturer's guide. FFPE tissue sections were probed with a mouse-specific transcriptome probe mix, followed by washing to remove unbound probes. Sequencing libraries were generated and assessed for quality. Finally, the libraries were sequenced on the Novaseq platform, and raw data were processed with the 10X Genomics Space Ranger pipeline.Spatial samples were integrated following the Seurat data integration pipeline (version 4.3.0)10. The number of features to return was set to 3000 in the "SelectIntegrationFeatures", with "SCT" as the normalization method and "CCA" (canonical correlation analysis) as the reduction. Differential gene expression (DE) was performed on spatial samples using the entire tissue slide to identify differences in gene expression between datasets. Pathway analysis was performed with pathfindR (version 1.6.4) using the DE output to further compare datasets, with KEGG as the gene set and using the greedy search algorithm to perform the active subnetwork search. Clustering was done on the spatial samples to annotate the spatial spots with a resolution value of 5[3].

Isolation and immunocytochemistry of primary murine Hepatic Stellate Cells

Livers of 7-8 months old WT and *Malrd1* KO mice were perfused with pronase and collagenase to isolate primary hepatic stellate cells (HSCs) at the Southern California Research Center for ALPD and Cirrhosis. Isolated HSCs were cultured *in vitro* for eight days, followed by immunocytochemistry on the six-well culture plates. After fixation and permeabilization, we incubated the cells with primary antibodies alpha-Smooth Muscle Actin (αSMA, MAB1420, Novus Biologicals, Minneapolis, MN) and MALRD1 (PA5-65203, Invitrogen, Waltham, Massachusetts) overnight. We used anti-mouse Alexa fluor 488 (A32766, Invitrogen, Waltham, Massachusetts) and anti-rabbit Alexa fluor 647 (A21443, Invitrogen, Waltham, Massachusetts) for detection. We used DAPI dye for nuclear counter stain (564907, BD Pharmingen, Franklin Lakes, NJ). We used Zeiss AxioObserver 7 (Carl Zeiss AG, Oberkochen, Germany) for image acquisition and Zeiss Zen software version 3.11 for image processing.

T0688 TGFβ1 treatment

We cultured T0688 (immortalized murine HSCs, Applied Biological Materials Inc., Richmond, BC, Canada) with basal media (BM) (DMEM+10% FBS) and TGFβ1 supplemented media at concentrations- 5ng/ml, 20ng/ml and 40ng/ml of murine recombinant transforming growth factor β1 (rTGFβ1, 7666-MB, R&D Systems, Minneapolis, MN) for six days in 12 well culture plates (Corning, Berlin, Germany). After six days, total RNA was isolated using the RNeasy Mini kit (Qiagen, Germany We have performed three independent experiments (N = 3) to determine the effect of TGFβ1 on *Malrd1* expression in T0688.

Immunocytochemistry of T0688

We cultured T0688 (immortalized murine hepatic stellate cells) with basal media (BM) (DMEM+10% FBS) and 40ng/ml of murine recombinant transforming growth factor β1 (rTGFβ1, 7666-MB, R&D Systems, Minneapolis, MN) for six days in polylysine-coated chamber slides (Corning, Berlin, Germany). After six days, we fixed and permeabilized the cells. We incubated the cells with primary antibodies against α-SMA and MALRD1 overnight. For detection, we have used anti-mouse Alexa Fluor 488 and anti-rabbit Alexa Fluor 647. We used DAPI dye for the nuclear counter stain. We used Leica Stellaris 5 white light laser (Leica Microsystems Inc., Deerfield, IL) for image acquisition and LAS X software for image processing.

Statistical Analysis

Statistical comparison between >2 experimental groups was performed using one-way or two-way ANOVA and post-hoc Bonferroni's test. Student's t-test was used in experiments with only two groups. A p-value of <0.05 was considered statistically significant. Results are presented as mean ± SEM.

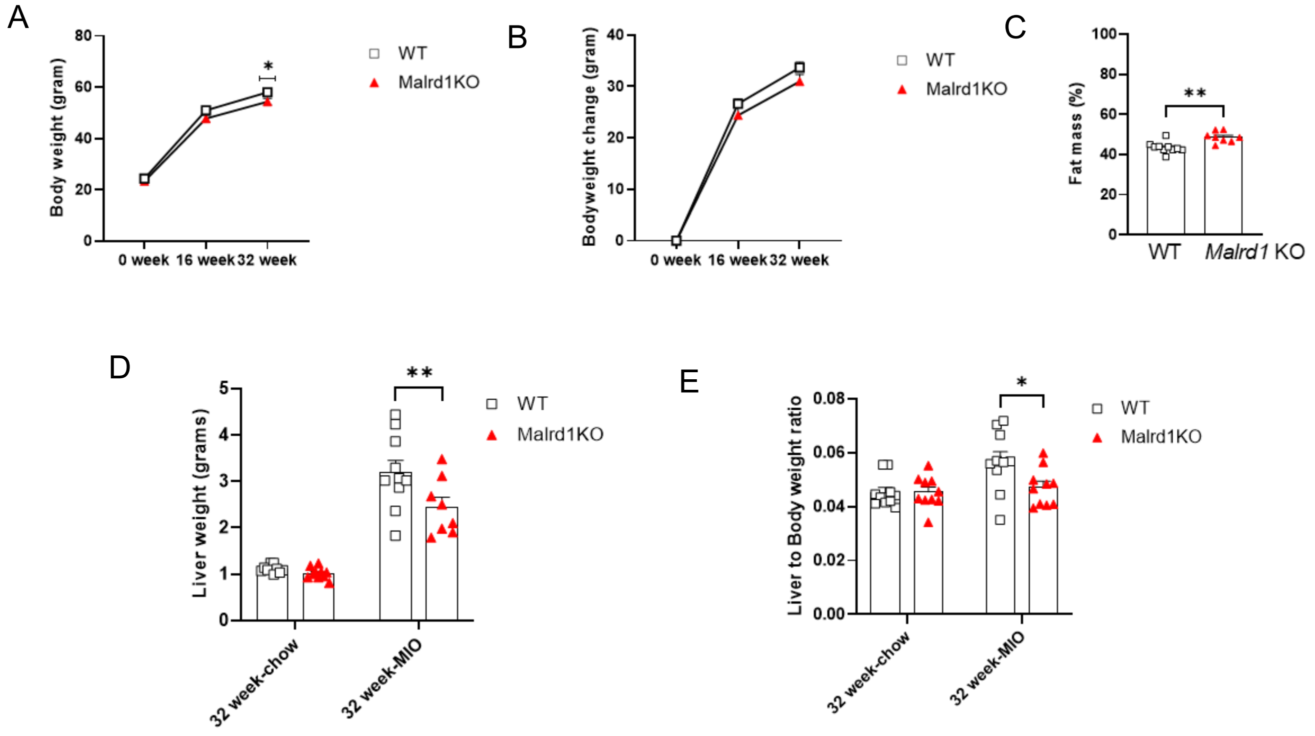

Supplementary Data

Supplementary Figure 1. 6-8 weeks old male wild type (WT) and *Malrd1* KO mice fed a MASH-inducing obesogenic (MIO) diet for 32 weeks *ad libitum*. (A) MIO-fed *Malrd1* KO mice have lower weight than WT mice, (B) No difference in the body weight change of MIO-fed *Malrd1* KO and WT mice at week 16 and week 32, (C) on MIO diet *Malrd1* KO have higher adiposity at week 32 than WT mice, (D) Chow fed WT and *Malrd1* KO mice have similar liver weight at week 32, interestingly MIO-fed *Malrd1* KO mice have lower liver weight than WT mice at week 32, (E) MIO-fed *Malrd1* KO mice have lower liver-to-body weight ratio than WT mice at week 32. (N=8-10). Mean± SEM. **P< 0.01, *P<0.05.

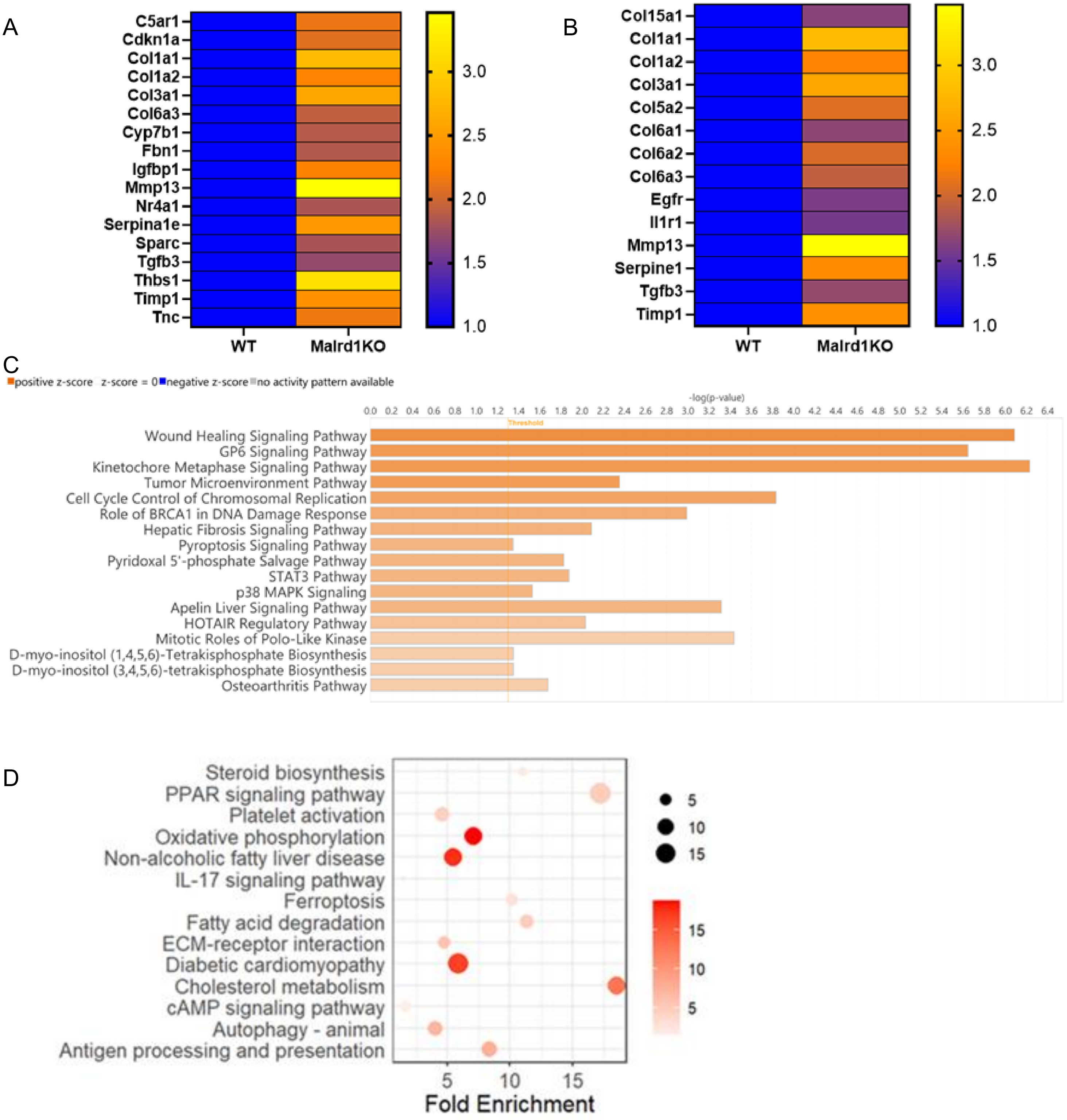

Supplementary Figure 2. 6-8 weeks old male wild type (WT) and *Malrd1* KO mice fed a MASH-inducing obesogenic (MIO) diet for 32 weeks *ad libitum*. (A) Heat map of differential expression genes (> 1.5 fold) demonstrating that the *Malrd1* KO mice have higher expression of hepatic fibrosis genes than WT mice, (B) Heat map of differential expression genes (> 1.5 fold) demonstrating that *Malrd1* KO mice have higher expression of genes responsible for hepatic stellate cell activation than WT mice, (C) Liver RNA Sequencing

data of *Malrd1* KO mice, when fed an MIO diet for 32 weeks, have higher expression of genes (positive z-score) involved in Wound Healing Signaling pathway, Hepatic Fibrosis Signaling Pathway and Pyroptosis Signaling Pathway in the liver than in the WT mice (N=5), (D) Spatial transcriptomics data of the liver cross section of *Malrd1* KO mice (N=3) revealed fold enrichment of pathways associated with hepatic stress like ferroptosis and oxidative phosphorylation compared to WT mice.

References

1. Bhattacharjee J, et.al. Hepatol Commun 2023;7:e0323.
2. Huttman M, et.al. Cochrane Database Syst Rev 2024; CD011929.
3. Rocque B, et.al. Scientific Reports 2024; 14:1 2024;14:1–15.
