# Supplementary material for: MAM and LDL Receptor Class A Domain Containing 1 Deficiency Aggravates Hepatic Fibrosis in Diet-Induced Metabolic Dysfunction-Associated Steatohepatitis
Source: Gastro Hep Adv. 2025 Nov 29;5(2):100854. doi: 10.1016/j.gastha.2025.100854 (PMC12805096; doi:10.1016/j.gastha.2025.100854)
Supplement: Extended PDF [file mmc2.pdf]

Gastro Hep Advances 2026;5:100854

RESEARCH LETTERS

MAM and LDL Receptor Class A Domain Containing 1 Deficiency Aggravates Hepatic Fibrosis in Diet-Induced Metabolic Dysfunction-Associated Steatohepatitis

Metabolic dysfunction-associated steatohepatitis (MASH) is the second leading cause of liver transplantation in the United States, posing a tremendous economic burden on health care.[1] Bile acid signaling, specifically farnesoid X receptor (FXR) agonists, has been investigated as a potential target for MASH therapeutics. Downstream of FXR activation, fibroblast growth factor 15/19 (FGF15/19; FGF15 in mice and FGF19 in humans) is secreted into the portal circulation, inhibiting bile acid synthesis and de novo lipogenesis, thereby reducing hepatic steatosis, inflammation, and hepatic fibrosis.[2] However, due to concerns for side effects such as dyslipidemia, FXR agonists have not received regulatory approval for the treatment of MASH.[3]

Previously, we demonstrated that whole-body *Fgf15*-deficient mice are resistant to a MASH-inducing obesogenic (MIO) diet.[4] MALRD1 (MAM and LDL receptor class A domain containing 1), also known as DIET1, ostensibly restricted to the intestinal epithelium, regulates the expression and secretion of FGF15/19 from the enterocytes, and variants of *MALRD1* have been shown to alter the expression and secretion of FGF19 from intestinal epithelial cells.[5] Interestingly, genome-wide association studies have shown associations of single-nucleotide polymorphisms in *MALRD1* with obesity-related traits.[6,7] Thus, considering *MALRD1* as a novel and understudied molecule in the bile acid signaling pathway, we investigated the role of MALRD1 in MASH progression using *Malrd1* knockout mice (*Malrd1* KO). Here we report the hitherto unknown presence of MALRD1 in hepatic stellate cells (HSCs) and outline MALRD1's FGF15/19 independent role in HSC-activated transforming growth factor β (TGFβ)-mediated MASH fibrosis.

Malrd1 KO gained body weight (Figure A1A and B) and adiposity (Figure A1C), unlike the whole-body *Fgf15* KO fed an MIO diet.[4] Plasma alanine aminotransferase levels in the *Malrd1* KO mice were higher on the MIO Diet (Figure 1A). Interestingly, *Malrd1* KO mice also had lower liver weight and liver-to-body weight ratios than wild-type mice on the MIO diet (Figure A1D and E). This phenotype of a smaller, "shrunken" liver matched the observation that Sirius Red staining of liver sections from *Malrd1* KO mice (Figure 1B). The *Malrd1* KO mice have a higher expression of TGFβ target genes in the liver (Figure 1C) and a higher incidence of advanced-stage fibrosis (Fibrosis Stage $>$ 2) (Figure 1D). The liver RNASeq data showed increased expression of genes (Fold change $>$ 1.5) involved in hepatic fibrosis and HSC activation in *Malrd1* KO mice (Figure A2A and B). Further pathway analysis of hepatic genes (1.5-fold upregulated and downregulated) revealed enrichment of signaling pathways, including the hepatic fibrosis signaling pathway (Figure A2C) in the *Malrd1* KO mice. Spatial transcriptomics using paraffin-embedded liver blocks from the Sirius Red fibrosis analysis revealed differential gene expression, with 18 distinct clusters found through neighborhood analysis (Figure 1E and F). Based on gene expression in liver sections, *Malrd1* deficiency induces metabolic stress pathways, such as ferroptosis and oxidative phosphorylation, which are known to be aggravated by TGFβ[8] (Figure A2D). Since we observed a higher incidence of hepatic fibrosis and activation of the hepatic fibrosis signaling pathway in the livers of *Malrd1* KO mice, we investigated the presence of MALRD1 in HSCs, a key player in the development of TGFβ-driven hepatic fibrosis. We confirmed the presence of MALRD1 in primary HSCs isolated from the wild-type mice livers (Figure 2A). Furthermore, we observed an increased expression of *Malrd1* in T0688 (immortalized murine HSC) cells treated with recombinant transforming growth factor-β1 (Figure 2B and C).

Our findings demonstrate an FGF15/19 independent role of MALRD1 in liver fibrosis, as unlike small intestine-specific *Fgf15* knockout mice,[9] *Malrd1* KO mice exhibit an obesogenic phenotype with a higher incidence of advanced liver fibrosis. We report for the first time that MALRD1 is present in HSCs and TGFβ1 induces its expression in HSCs. Our data suggests a possible function of MALRD1 in impeding TGFβ-mediated fibrosis signaling, wherein the absence of MALRD1 aggravates the expression of TGFβ target genes, leading to fibrosis, and a higher TGFβ1 concentration induces *Malrd1* expression in the HSC, suggesting the importance of MALRD1 in restricting HSC activation, which is essential for reversing fibrosis.[10] Since we used whole-body *Malrd1* KO mice, our study could not distinguish the contribution of HSC-MALRD1 versus intestinal-MALRD1 to MASH pathogenesis. In the future, studies using tissue-specific *Malrd1*-deficient mice will provide insight into the molecular interactions of

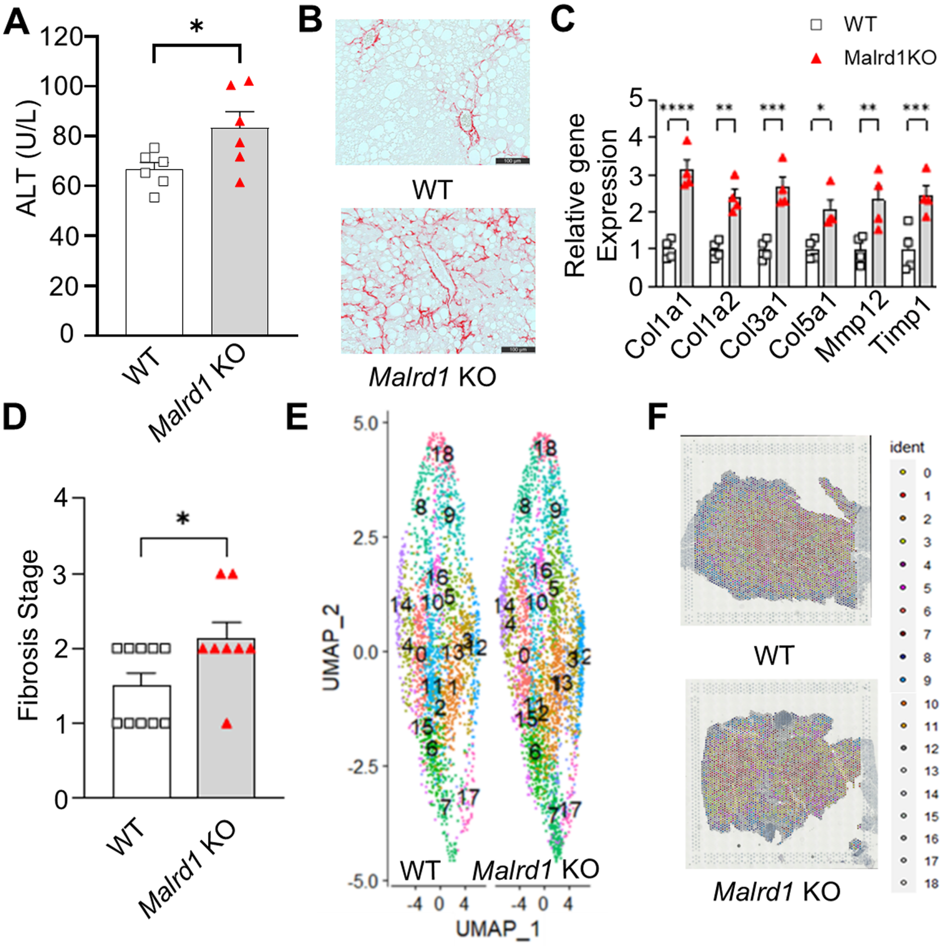

Figure 1. *Malrd1* KO mice exhibit higher Alanine transaminase and aggravated hepatic fibrosis. 6–8 weeks old male wild-type (WT) and *Malrd1* KO mice fed a MASH-inducing obesogenic (MIO) diet for 32 weeks *ad libitum*. (A) MIO-fed *Malrd1* KO have a higher plasma alanine transaminase (ALT) (U/L) levels than WT mice at week 32, (B) Representative histology image of Sirius Red staining of the liver cross-section from WT and Malrd1KO mice, (C) *Malrd1* KO mice higher expression of transforming growth factor β (TGFβ) target genes in the liver than WT mice, (D) *Malrd1* KO mice group has a higher incidence of advanced-stage hepatic fibrosis than WT mice ($P < .05$), (E) Representative image of spatial transcriptomics neighborhood cluster based on the differential expression of genes, (F) Spatial distribution of the clusters across the liver cross-section of MIO-fed WT and *Malrd1* KO mice. (N = 4-10). t-test or Two-Way Anova. Mean ± SEM. ****$P < .0001$, ***$P < .001$, **$P < .01$, *$P < .05$.

MALRD1 in the HSC during MASH and the influence of intestinal MALRD1 on the action of HSC-MALRD1 during MASH that might lead to the identification of potential therapeutic targets for MASH.

JASHDEEP BHATTACHARJEE[1]
LINDA X. WANG[1]
BRIANNA MENESES[1]
JULIET A. EMAMAULLEE[2]
MARK R. FREY[1,3]
ROHIT KOHLI[1]

[1]Department of Pediatrics, Division of Gastroenterology, Hepatology and Nutrition, Children's Hospital Los Angeles, Los Angeles, California

[2]Division of Abdominal Organ Transplantation and Hepatobiliary Surgery, Department of Surgery, Keck School of Medicine, University of Southern California, Los Angeles, California

[3]Department of Cancer Biology, Keck School of Medicine, University of Southern California, Los Angeles, California

Correspondence:
Address correspondence to: Jashdeep Bhattacharjee, PhD, Children's Hospital Los Angeles, Department of Pediatrics, Division of Gastroenterology, Hepatology and Nutrition, 4650 Sunset Blvd, Los Angeles, California 90027. e-mail: jbhattacharjee@chla.usc.edu.

Supplementary Material

Supplementary material associated with this article can be found, in the online version, at https://doi.org/10.1016/j.gastha.2025.100854.

References

1. Tapper EB, et al. J Med Econ 2023;26:348–356.
2. Inagaki T, et al. Cell Metab 2005;2:217–225.
3. Kremoser C. J Hepatol 2021;75:12–15.
4. Myronovych A, et al. Am J Physiol Gastrointest Liver Physiol 2020;319:G669–G684.
5. Lee JM, et al. J Lipid Res 2018;59:429–438.

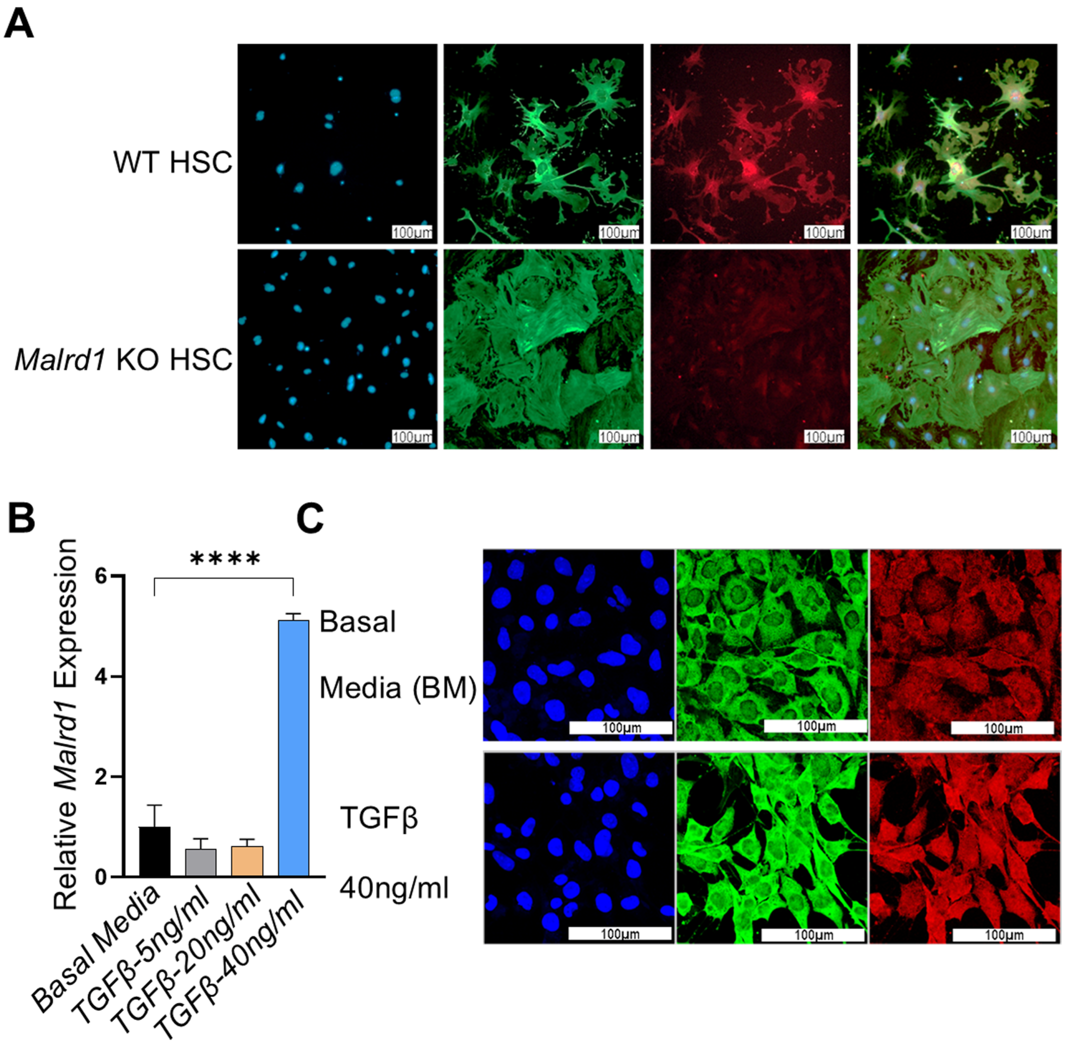

Figure 2. Activated HSCs express MALRD1. (A) Primary murine HSC isolated from WT mice expressed MALRD1 (red) and α-smooth muscle actin (αSMA, green). HSCs from *Malrd1* KO mice express αSMA but not MALRD1. Immortalized murine hepatic stellate cells (T0688) were treated with recombinant murine transforming growth factor-β1 (TGFβ1) at 5 ng/ml, 20 ng/ml, and 40 ng/ml for 6 days. At a 40 ng/ml concentration, T0688 has shown (B) higher expression of *Malrd1* and (C) brighter staining for MALRD1 (red) than T0688 in basal media. Nucleus counterstain DAPI (Blue). (N = 3), One-way Anova, Mean ± SEM. ****$P < .0001$.

6. Gurung RL, et al. J Diabetes Res 2020;2020:5016916.
7. Comuzzie AG, et al. PLoS One 2012;7:e51954.
8. Kim DH, et al. Cell Death Dis 2020; 11:1–13.
9. Bozadjieva-Kramer N, et al. JCI Insight 2024;9:e174164.
10. De Smet V, et al. Cell Death Dis 2021;12:1–10.

Abbreviations used in this paper: **FGF15/19, fibroblast growth factor 15/19; FXR, farnesoid X receptor; HSCs, hepatic stellate cells; *Malrd1* KO, *Malrd1* knockout mice; MALRD1, MAM and LDL receptor class A domain containing 1; MASH, metabolic dysfunction-associated steatohepatitis; MIO, MASH-inducing obesogenic; TGFβ, transforming growth factor β.**

Most current article

Copyright © 2025 The Authors. Published by Elsevier Inc. on behalf of the AGA Institute. This is an open access article under the CC BY license (http://creativecommons.org/licenses/by/4.0/).
2772-5723
https://doi.org/10.1016/j.gastha.2025.100854

Received October 31, 2025. Accepted November 26, 2025.

Acknowledgments:
The authors thank Prof. Karen Reue of the Department of Human Genetics, David Geffen School of Medicine, University of California, Los Angeles, for providing the whole body Malrd1 knockout mice used in this study. The authors also thank Einar Thor Hafberg of SEHA Sheikh Khalifa Medical City and Mikako Warren of the Department of Pathology at Children's Hospital Los Angeles for their valuable scientific advice. The authors acknowledge the Spatial Biology and Genomics Core, Cellular Imaging Core, and Stem Cell Core of the Saban Research Institute at Children's Hospital Los Angeles, as well as the Integrative Liver Cell Core of University of Southern California, for their technical support.

Conflicts of Interest:
The authors disclose no conflicts.

Funding:
The study was supported by the CHLA Pilot Research grant (JB), NASPGHAN Foundation Nestlé Nutrition Research Young Investigator Development Award (LXW), the CHLA 2nd NIH R01 Pilot grant, and National Institutes of Health award R01DK100314 (RK).

Ethical Statement:
The Institutional Animal Care and Use Committee (IACUC) of Children's Hospital Los Angeles (assurance#D16-00175) has approved the animal research protocol for the study. We have no human data in this study.

Data Transparency Statement:
Data may be made available to other researchers upon request to the corresponding author.

Reporting Guidelines:
None.

Gastro Hep Advances, Volume 5

Supplemental information

MAM and LDL Receptor Class A Domain Containing 1 Deficiency Aggravates Hepatic Fibrosis in Diet-Induced Metabolic Dysfunction-Associated Steatohepatitis

Jashdeep Bhattacharjee, Linda X. Wang, Brianna Meneses, Juliet A. Emamaullee, Mark R. Frey, and Rohit Kohli

Materials and methods

All animal studies were reviewed, approved, and monitored by Animal Care and Use Committee at Children's Hospital Los Angeles (assurance#D16-00175). 6-8 week-old male C57Bl6/J wild type (WT) and whole body *Malrd1* knockout (*Malrd1* KO) mice were housed in a 12-hour light-dark cycle maintained in a (22±2˚C) temperature-controlled room. Beginning at 6 to 8 weeks of age, male WT and *Malrd1* KO mice were fed on a MASH-inducing obesity (MIO) diet (D12331i, Research Diets, Inc., 58 kcal % fat; Research Diets, New Brunswick, NJ) and drinking water containing fructose (55% fructose by weight; Acros Organics, Morris Plains, NJ) and sucrose (45% sucrose by weight; Acros Organics, Morris Plains, NJ) mixture at a concentration of 42 g/l[1]. Animals were provided *ad libitum* access to the MIO diets.

Body composition

The body composition of mice (adiposity) was determined using an EchoMRI™-100H Body Composition Analyzer (EchoMRI, Texas, USA).

Plasma alanine transaminase (ALT) assay

Plasma isolated from whole blood was used to measure ALT concentration using the ALT Activity Assay kit (Sigma, St. Louis, MO) according to the manufacturer's instructions.

Liver Histology Analysis

Sirius Red staining was used to evaluate hepatic fibrosis stage (0–4)[2].

qPCR

5µg of total RNA was used to generate cDNA using the SuperScript III First-Strand Synthesis System (Invitrogen, Carlsbad, CA). Quantitative PCR was performed with the Taqman Probe using TaqMan™ Fast Advanced Master Mix (Applied Biosystems, Foster City, CA) in the 7900HT qPCR platform (Applied Biosystems, Foster City, CA). ΔΔCt was used to calculate the fold change of expression of *Malrd1* (PrimerSequence:Forward 5'-CAGAACCCTCAGTAGAAAGGTTAG 3'; Reverse 5' TGGTGGCACTTGTGTGATAG-3',Probe 5' /56-FAM/AGGATGGAC/ZEN/AGGAAATCGATGCCA/3IABkFQ 3'), *Col1a1, Col1a2, Col3a1, Col5a1, Mmp12, Timp1* normalized with the housekeeping gene, *Rpl18* using Taqman probes (Thermo Fisher Scientific, Carlsbad, CA).

RNA Sequencing

5µg of total RNA from the livers of WT and *Malrd1* KO mice (N=5) were sequenced on the Illumina NextSeq platform. The reads were mapped to the UCSC transcript set, and gene expression was estimated using RSEM. Differentially expressed genes were identified with edgeR. QIAGEN IPA was used for pathway analysis.

Spatial Transcriptomics

Spatial transcriptomic profiling was conducted using the 10× Genomics Visium CytAssist on liver sections from one wild-type and three *Malrd1* knockout mice. RNA was extracted with the Qiagen RNeasy kit, and quality was checked using an Agilent BioAnalyzer. Samples were prepared and sectioned onto charged slides, then processed according to the manufacturer's guide. FFPE tissue sections were probed with a mouse-specific transcriptome probe mix, followed by washing to remove unbound probes. Sequencing libraries were generated and assessed for quality. Finally, the libraries were sequenced on the Novaseq platform, and raw data were processed with the 10X Genomics Space Ranger pipeline.Spatial samples were integrated following the Seurat data integration pipeline (version 4.3.0)10. The number of features to return was set to 3000 in the "SelectIntegrationFeatures", with "SCT" as the normalization method and "CCA" (canonical correlation analysis) as the reduction. Differential gene expression (DE) was performed on spatial samples using the entire tissue slide to identify differences in gene expression between datasets. Pathway analysis was performed with pathfindR (version 1.6.4) using the DE output to further compare datasets, with KEGG as the gene set and using the greedy search algorithm to perform the active subnetwork search. Clustering was done on the spatial samples to annotate the spatial spots with a resolution value of 5[3].

Isolation and immunocytochemistry of primary murine Hepatic Stellate Cells

Livers of 7-8 months old WT and *Malrd1* KO mice were perfused with pronase and collagenase to isolate primary hepatic stellate cells (HSCs) at the Southern California Research Center for ALPD and Cirrhosis. Isolated HSCs were cultured *in vitro* for eight days, followed by immunocytochemistry on the six-well culture plates. After fixation and permeabilization, we incubated the cells with primary antibodies alpha-Smooth Muscle Actin (αSMA, MAB1420, Novus Biologicals, Minneapolis, MN) and MALRD1 (PA5-65203, Invitrogen, Waltham, Massachusetts) overnight. We used anti-mouse Alexa fluor 488 (A32766, Invitrogen, Waltham, Massachusetts) and anti-rabbit Alexa fluor 647 (A21443, Invitrogen, Waltham, Massachusetts) for detection. We used DAPI dye for nuclear counter stain (564907, BD Pharmingen, Franklin Lakes, NJ). We used Zeiss AxioObserver 7 (Carl Zeiss AG, Oberkochen, Germany) for image acquisition and Zeiss Zen software version 3.11 for image processing.

T0688 TGFβ1 treatment

We cultured T0688 (immortalized murine HSCs, Applied Biological Materials Inc., Richmond, BC, Canada) with basal media (BM) (DMEM+10% FBS) and TGFβ1 supplemented media at concentrations- 5ng/ml, 20ng/ml and 40ng/ml of murine recombinant transforming growth factor β1 (rTGFβ1, 7666-MB, R&D Systems, Minneapolis, MN) for six days in 12 well culture plates (Corning, Berlin, Germany). After six days, total RNA was isolated using the RNeasy Mini kit (Qiagen, Germany We have performed three independent experiments (N = 3) to determine the effect of TGFβ1 on *Malrd1* expression in T0688.

Immunocytochemistry of T0688

We cultured T0688 (immortalized murine hepatic stellate cells) with basal media (BM) (DMEM+10% FBS) and 40ng/ml of murine recombinant transforming growth factor β1 (rTGFβ1, 7666-MB, R&D Systems, Minneapolis, MN) for six days in polylysine-coated chamber slides (Corning, Berlin, Germany). After six days, we fixed and permeabilized the cells. We incubated the cells with primary antibodies against α-SMA and MALRD1 overnight. For detection, we have used anti-mouse Alexa Fluor 488 and anti-rabbit Alexa Fluor 647. We used DAPI dye for the nuclear counter stain. We used Leica Stellaris 5 white light laser (Leica Microsystems Inc., Deerfield, IL) for image acquisition and LAS X software for image processing.

Statistical Analysis

Statistical comparison between >2 experimental groups was performed using one-way or two-way ANOVA and post-hoc Bonferroni's test. Student's t-test was used in experiments with only two groups. A p-value of <0.05 was considered statistically significant. Results are presented as mean ± SEM.

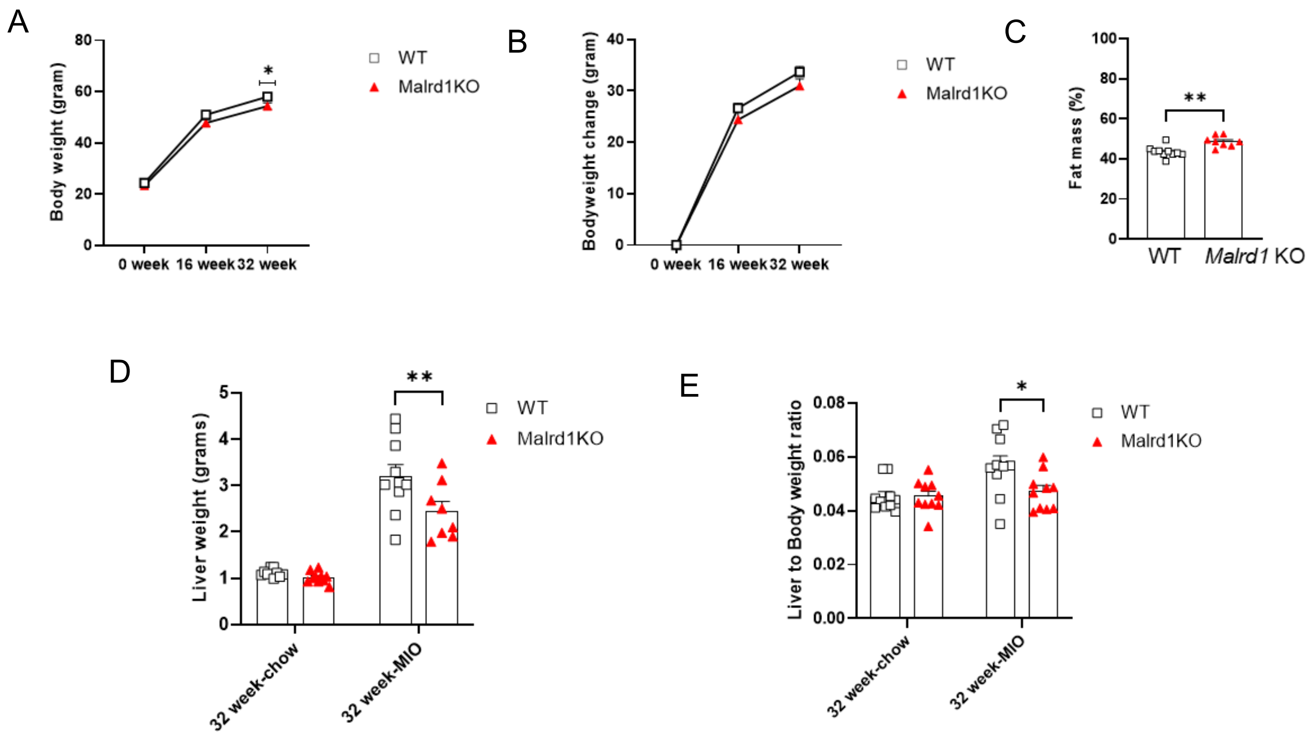

Supplementary Data

Supplementary Figure 1. 6-8 weeks old male wild type (WT) and *Malrd1* KO mice fed a MASH-inducing obesogenic (MIO) diet for 32 weeks *ad libitum*. (A) MIO-fed *Malrd1* KO mice have lower weight than WT mice, (B) No difference in the body weight change of MIO-fed *Malrd1* KO and WT mice at week 16 and week 32, (C) on MIO diet *Malrd1* KO have higher adiposity at week 32 than WT mice, (D) Chow fed WT and *Malrd1* KO mice have similar liver weight at week 32, interestingly MIO-fed *Malrd1* KO mice have lower liver weight than WT mice at week 32, (E) MIO-fed *Malrd1* KO mice have lower liver-to-body weight ratio than WT mice at week 32. (N=8-10). Mean± SEM. **P< 0.01, *P<0.05.

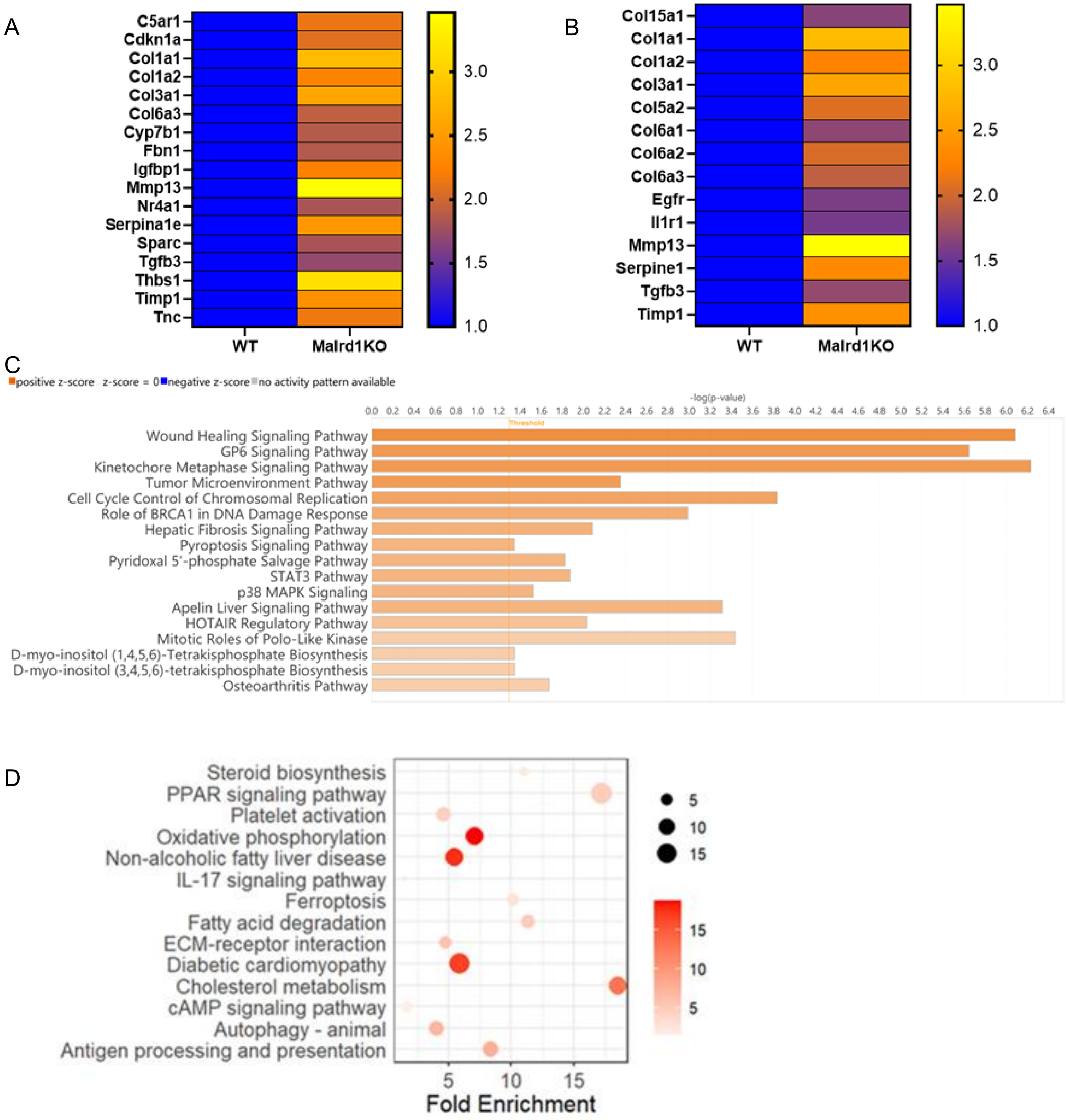

Supplementary Figure 2. 6-8 weeks old male wild type (WT) and *Malrd1* KO mice fed a MASH-inducing obesogenic (MIO) diet for 32 weeks *ad libitum*. (A) Heat map of differential expression genes (> 1.5 fold) demonstrating that the *Malrd1* KO mice have higher expression of hepatic fibrosis genes than WT mice, (B) Heat map of differential expression genes (> 1.5 fold) demonstrating that *Malrd1* KO mice have higher expression of genes responsible for hepatic stellate cell activation than WT mice, (C) Liver RNA Sequencing

data of *Malrd1* KO mice, when fed an MIO diet for 32 weeks, have higher expression of genes (positive z-score) involved in Wound Healing Signaling pathway, Hepatic Fibrosis Signaling Pathway and Pyroptosis Signaling Pathway in the liver than in the WT mice (N=5), (D) Spatial transcriptomics data of the liver cross section of *Malrd1* KO mice (N=3) revealed fold enrichment of pathways associated with hepatic stress like ferroptosis and oxidative phosphorylation compared to WT mice.

References

1. Bhattacharjee J, et.al. Hepatol Commun 2023;7:e0323.
2. Huttman M, et.al. Cochrane Database Syst Rev 2024; CD011929.
3. Rocque B, et.al. Scientific Reports 2024; 14:1 2024;14:1–15.
